# Identification and functional characterisation of a *Schistosoma japonicum* insulin-like peptide

**DOI:** 10.1186/s13071-017-2095-7

**Published:** 2017-04-14

**Authors:** Xiaofeng Du, Donald P. McManus, Pengfei Cai, Wei Hu, Hong You

**Affiliations:** 1 Molecular Parasitology Laboratory, Infectious Diseases Division, QIMR Berghofer Medical Research Institute, Brisbane, Queensland Australia; 2 School of Life Sciences, Fudan University, 2005 Songhu Road, Shanghai, 200438 China; 3 National Institute of Parasitic Diseases, Chinese Center for Disease Control and Prevention, 207 Ruijin Er Road, Shanghai, 200025 China

**Keywords:** Schistosome, Insulin-like peptide, Insulin receptor

## Abstract

**Background:**

Previous studies have shown that insulin receptors in schistosomes, triggered by host insulin, play an important role in parasite growth, development and fecundity by regulating glucose metabolism. However, limited information is available on the recently identified endogenous insulin-like peptide (ILP) in blood flukes.

**Results:**

We isolated ILPs from *Schistosoma japonicum* (*Sj*ILP) and *S. recogni*sed (*Sm*ILP) and present results of their molecular and structural analysis. *Sj*ILP shares 63% amino acid identity with *Sm*ILP, but only 18% identity with human insulin. There is high cross immunological reactivity between the *S. japonicum* and *S. mansoni* ILPs as observed in western blots using an anti-*Sj*ILP polyclonal antibody. ADP binding/hydrolysis ability was observed in both *Sj*ILP and *Sm*ILP, but not in human insulin, suggesting a parasite-specific role for ILP compared with host insulin. Protein binding assays using the Octet-RED system showed *Sj*ILP binds *S. japonicum* IRs (*Sj*IR1 and *Sj*IR2) strongly. An anti-phospho antibody against extracellular signal-regulated kinase (Erk) recognised a 44-kDa target band in an extract of adult worms after stimulation by r*Sj*ILP in vitro*,* suggesting an important role for *Sj*ILP in activating *Sj*IRs and in regulating downstream signal transduction. Immunolocalisation showed *Sj*ILP is located on the tegument and the underlying musculature, similar to that observed for *Sj*IR1, but it is also present throughout the parenchyma of males and in the vitelline cells of females, the same locations as *Sj*IR2 described in an earlier published study of ours*.* The same localisation of *Sj*ILP and the *Sj*IRs is suggestive of an interaction between the insulin-like peptide and the IRs. In addition to binding host insulin, schistosomes also can express their own endogenous ILPs, which can activate the parasite insulin signal pathway, thereby playing a critical role in worm growth, development and fertility.

**Conclusions:**

These findings shed new light on ILPs in schistosomes, providing further insight into the distinct and specialised functions of *Sj*IR1 and 2 in *S. japonicum* and their interaction with host insulin.

**Electronic supplementary material:**

The online version of this article (doi:10.1186/s13071-017-2095-7) contains supplementary material, which is available to authorized users.

## Background

The life-cycle of schistosomes involves snail intermediate hosts and definitive mammalian hosts including humans, in whom schistosomes can survive for decades [1]. Remarkably, the highly sophisticated relationship between schistosomes and their mammalian hosts seems to involve exploitation by these parasites of host endocrine and immune signals [2, 3]. By binding many ligands including hormones, growth factors, cytokines, receptor tyrosine kinases (RTKs), which are high-affinity cell surface receptors, can be activated to trigger different signalling cellular cascades for the regulation of cell proliferation, differentiation and survival [4]. Numerous data have demonstrated that schistosome RTKs can bind host growth factors, activating conserved kinase signalling pathways which are involved in worm growth and development [5]. RTK signalling is active in the reproductive organs of schistosomes where it likely regulates gametogenesis, sexual maturation and egg production [6]. Given the fact that schistosome eggs play key roles in both parasite transmission and pathogenesis in mammalian hosts, RTKs and their signalling partners represent potentially important targets for chemotherapeutic attack. Genome-wide searches have recently reported the presence of an insulin signalling pathway (a typical RTK signal pathway) in *S. japonicum* [7], *S. mansoni* [8] and *S. haematobium* [9, 10]. Further, classic downstream components of the insulin pathway have been identified in schistosomes, including Src homology-containing (SHC), Src homology 2-B proteins, phosphoinositide-3-kinase (PI3K), extracellular signal-regulated kinase (ERK), glycogen synthase (GYS) and glucose transport protein 4 (GTP4) [7–11]. Previous studies have shown that host insulin can stimulate the metabolism, growth and development of adult [12] and larval schistosomes [13] by increasing glucose uptake and enhancing the survival of schistosome larvae [14]. Moreover, the presence of human insulin increases the glucose content of adult male and female *S. japonicum* 1.7- and 2.9-fold, respectively, in vitro [15], suggesting insulin plays a key role in regulating glucose uptake in this parasite. Our previous microarray analysis also demonstrated that host insulin plays an important role in insulin signalling in schistosomes by stimulating glucose metabolism through up-regulation of the phosphoinositide-3-kinase (PI3K) sub-pathway [15]. In addition, host insulin is also necessary for worm fecundity due to its activation of the mitogen-activated protein kinases (MAPK)/ERK sub-pathway [15], thereby regulating cell proliferation, differentiation and survival. Two types of insulin receptors have been isolated from *S. mansoni* (*Sm*IR-1 and *Sm*IR-2) [16] and *S. japonicum* (*Sj*IR1 and *Sj*IR2) [17], which were shown to bind human insulin in two-hybrid analysis and the Octet-RED system [18]. In summary, these studies imply that insulin signalling, activated by host insulin, plays an important role in the growth, development and fecundity of schistosomes.

There have been 37 insulin-like ligands (ILPs) identified in free-living *Caenorhabditis. elegans* [19] but limited information is available on the presence and biological importance of insulin-like peptides (ILPs) in schistosomes and other platyhelminths. Genome-wide searches for ILPs have recently reported the presence of two ILPs in *Taenia* and *Echinococcus* spp*.* [20]. Moreover, in *Taenia solium*, one ligand, ILP-1, was shown to be predominantly expressed in ovarian tissues, suggesting an important role in worm fecundity [20]. However, to date, only one ILP has been identified in *S. japonicum* and *S. mansoni* [20], and there is no information to indicate how schistosomes utilise the ILP, and what its function might be in these parasites. In this study, we employed molecular structural analysis, protein interaction and phosphorylation assays, and used immunolocalisation procedures to explore the functional roles of ILPs in schistosomes.

## Methods

### Parasites

*Schistosoma japonicum* adult worms were collected by perfusion of female Animal Resource Centre (ARC) Swiss mice infected percutaneously with 60 cercariae (Anhui population, mainland China) shed from *Oncomelania hupensis hupensis* snails as described [21] at the QIMRB animal facility. *S. mansoni* adult worms were collected by perfusion of ARC Swiss mice infected percutaneously with 160 cercariae shed from *Biomphalaria glabrata* snails [22]. Infected *B. glabrata* snails were kindly provided by the NIAID Schistosomiasis Resource Centre, Biomedical Research Institute, Rockville, Maryland, USA.

### Cloning insulin-like peptides (ILP) from *S. japonicum, S. mansoni*

A Qiagen RNeasy kit (Qiagen, Hilden, Germany) was used to purify total RNA from adult *S. japonicum* and *S. mansoni.* A one-step RT-PCR (Qiagen) kit was employed to amplify specific cDNA. Based on a sequence in GenBank for an ILP in *S. japonicum* (AY814982), primers for *Sj*ILP were designed (Additional file 1: Table S1) to obtain the full-length cDNA. Using BLAST on the *Sj*ILP sequence in SchistoDB (http://schistodb.net/schisto), we obtained predicted genomic sequences for ILP in *S. mansoni* (*Sm*ILP), *S. haematobium* (*Sh*ILP) and designed appropriate primers for amplifying full-length cDNA sequences for *Sm*ILP (Additional file 1: Table S1).

### Sequence and structural analysis

Searches for homologous insulin-like peptide sequences were performed using BLAST on the NCBI website (http://blast.ncbi.nlm.nih.gov/Blast.cgi) and the WormBase ParaSite website (http://parasite.wormbase.org/Multi/Tools/Blast). Molecular weight and isoelectric point determinations were performed using the ExPASy-Compute pI/Mw tool (http://web.expasy.org/compute_pi/). Signal sequences were identified using the SignalP 4.1 server (http://www.cbs.dtu.dk/services/SignalP/) [23]. The PHYRE2 protein fold recognition server (http://www.sbg.bio.ic.ac.uk/phyre2/) was used to generate three-dimensional (3D) models [24] of the schistosome ILP, and binding site predictions were carried out using the 3DLigandSite (http://www.sbg.bio.ic.ac.uk/3dligandsite/) [25].

### Protein expression, purification and antibody generation

C-terminal fragments of *Sj*ILP (from T31 to S130) and *Sm*ILP (T32-N133) were amplified and cloned into the pET28b vector (Novagen, Madison, USA) using a forward primer with a *Bam*H I restriction site (underlined) and a reverse primer with a *Sal* I restriction site (underlined). Forward (5'-AAG GAT CCG ACA CAT AGT TTA CCA GAA TTA CAA A-3') and reverse (5'-AAG TCG ACG CTA GGA TTG CAA AAT TGT TCT-3') primers for *Sj*ILP and forward (5'- AAG GAT CCG ACA CAA ACT TTA ACA GAA TTG AAT AC-3') and reverse (5'- AAG TCG ACA TTT GGA TTA CAA AAT TGT TCT-3') primers for *Sm*ILP were used for amplification and cloning into the pET28b vector.

The reconstructed vectors were then transformed into *Escherichia coli* (BL21 strain) for expression induced with 1 mM IPTG (isopropyl thio-b-D-galactoside) at 37 °C for 3 h. Recombinant *Sj*ILP (r*Sj*ILP) and *Sm*ILP (r*Sm*ILP) proteins were purified from inclusion bodies by chromatography using a Ni-NTA His-tag affinity kit (Novagen) under denaturing conditions using 6 M guanidine according to the manufacturer’s instructions. Purified proteins were then refolded in buffer (55 mM Tris-HCl, 21 mM NaCl, 0.88 mM KCl, 2 mM reduced glutathione, 0.4 mM oxidized glutathione, 1 mM EDTA, 10% w/v sucrose, pH 8.2).

Polyclonal antibodies were raised against the *Sj*ILP fusion protein in 8 weeks old female Balb/c mice. Briefly, five mice were immunised three times each with 25 μg recombinant protein adjuvanted with Quil A (Superfos, Denmark) at 3-week intervals [26]. The immunisations were delivered by intraperitoneal injection. Blood was collected 2 weeks after the third injection. The titre of the antibody was determined using an enzyme-linked immunosorbent assay (ELISA). Briefly, Maxisorb immunoplates (Nalge Nune International, USA) were coated overnight at 4 °C with r*Sj*ILP protein (100 μl of 0.5 μg/ml) in coating buffer (100 μl/well). After three washes with 0.05% (v/v) Tween in PBS (PBST), wells were blocked with 200 μl of 5% (v/v) skim milk in PBS (SMP) and incubated for 1 h at 37 °C. The mouse anti-*Sj*ILP serum was serially diluted (from 1:200 to 1:102,400) in SMP, and 100 μl in duplicate of each dilution were added to individual wells. After incubation at 37 °C for 1 h, the wells were washed with PBST for three times and 100 μl (1:2,000 dilution) of horseradish peroxidise (HRP)-conjugated goat anti-mouse IgG (Invitrogen) was added. After incubation at 37 °C for 1 h, the wells were washed with PBST for five times, 100 μl of substrate solution [2,2-azino-di-(ethyl-benzithiozolin sulfonate)] (Sigma-Aldrich, Castle Hill, Australia) was added, the wells were then incubated at room temperature and read on a plate reader using Microplate manager software (Bio-Rad, Mississauga, Canada).

### Western blot analysis

The mouse anti-*Sj*ILP serum was used in Western blotting to probe electrophoresed proteins; these proteins included: 1) Purified recombinant *Sj*ILP and *Sm*ILP; 2) Tegument protein and residual carcass protein [27] extracted from adult *S. japonicum* freshly perfused from mice percutaneously infected with *S. japonicum* as described earlier; 3) Native *S. mansoni* protein extracts prepared [17] from adult worms freshly perfused from mice percutaneously infected with *S. mansoni*. An adult worm antigen preparation (SWAP) was made from schistosomes as described [17], after adult worms were washed in perfusion buffer (8.5 g NaCl and 15 g NaCitrate in 1 l of water) three times, to minimise contamination of the schistosome protein extract with host components. To determine whether there was any immunological cross-reactivity between human insulin (Sigma-Aldrich) and *Sj*ILP, the mouse anti-*Sj*ILP antibody, a mouse anti-human insulin monoclonal antibody (Abcam, Cambridge, UK) and naïve mouse sera as control were used in Western blotting to probe equal quantities of electrophoresed human insulin and r*Sj*ILP. The concentrations of all proteins used were determined using the Bio-Rad protein assay dye reagent (Bio-Rad).

Protein samples were separated on 15% (w/v) SDS-PAGE gels and transferred to an Immun-Blot low fluorescence-PVDF membrane. Overnight blocking was performed with Odyssey buffer containing 4% (v/v) goat serum at 4 °C. Then, the membrane was subjected to incubation with the mouse anti-*Sj*ILP or the anti-human insulin monoclonal antibody (diluted in Odyssey buffer and 0.1% (v/v) Tween-20) for 1 h followed by incubation with IRDye-labeled 680LT goat anti-mouse IgG antibody (Li-COR Biosciences) (1:15,000 diluted in Odyssey buffer with 0.1% Tween-20 and 0.01% SDS) for 1 h on a shaker in a dark chamber. After a final wash with distilled water, the membrane was allowed to dry in the dark and visualised using the Odyssey CLx Infrared Imaging System [22].

### Detection of mitogen-activated protein kinase (MAPK) using an anti-phospho-P44/42 MAPK (Erk1/2) antibody

It had been shown previously that an anti-phospho-P44/42 MAPK (Erk1/2) antibody could recognize exclusively activated (phosphorylated) forms of this kinase in *S. mansoni* [28] because of the high sequence homology in key phosphorylation sites between kinases from schistosomes and humans, and because phosphorylation at these sites is vital for enzyme activation [28]. Based on the fact that the p44/42 MAPK (Erk1/2) signalling pathway can be activated in response to extracellular stimuli including growth factors [29], an anti-phospho-P44/42 MAPK (Erk1/2) antibody (Cell Signaling Technology, New England Biolabs, Ipswisch, USA) was used to detect the presence of an Erk band in an extract of adult *S. japonicum* following the incubation of adult worms in the presence of human insulin (Sigma-Aldrich) or r*Sj*ILP. Briefly, freshly perfused adult *S. japonicum* worms were cultured over night at 37 °C in DMEM (Gibco, Waltham, USA); then the worms were incubated for 30 min in fresh DMEM containing: 1). r*Sj*ILP (1 μM); 2). r*Sj*ILP (1 μM) + human insulin (1 μM); 3). human insulin (1 μM).; and 4). neither r*Sj*ILP nor human insulin. Worms from the different groups were then collected for SWAP extraction which was undertaken in the presence of HALT protease/phosphatase inhibitor cocktail (ThermoFisher, Waltham, USA). SDS-PAGE/Western blotting was carried out as described above using the Odyssey system. Tyrosine kinase activity was detected using a mouse anti-phospho-P44/42 MAPK (Erk1/2) antibody (1:1,000) as primary antibody (1:1,000 dilution in Odyssey buffer and 0.1% Tween-20) for 1 h followed by incubation with IRDye-labeled 680LT goat anti-mouse IgG antibody (Li-COR Biosciences, Lincoln, USA) (1:15,000 diluted in Odyssey buffer with 0.1% Tween-20 and 0.01% SDS). An anti-actin antibody (Sigma-Aldrich) (1:150) [30] was used to assess protein-loading differences. OdysseyClassic 3.0 software was used for Western blot quantification.

### Immunolocalisation

Horseradish peroxidise (HRP) labelling was used for the immunolocalisation of *Sj*ILP in adult *S. japonicum* as described [27]. Freshly perfused male and female worms were fixed in 100% methanol, embedded in Tissue-Tek Optimal Cutting Temperature (OCT) compound (ProSciTech, Queensland, Australia), and 7.0 μm cryostat sections were produced. HRP labelling was performed according to standard procedures [17]. The primary antibody solution was a 1:100 dilution of the mouse anti-*Sj*ILP serum and normal mouse serum (1:100) was used as a control. Non-specific antibody binding was inhibited by incubating the sections in 10% (v/v) normal goat serum in PBS. ImmPRESS HRP Anti-mouse IgG (Peroxidase) Polymer (Vector Labs, California, USA) was used as a secondary antibody to detect immunolocalisation. Slides were scanned and digitised using a ScanScope XT (Aperio, California, USA).

HRP labelling was also used with a mouse anti-human insulin monoclonal antibody (Abcam, Cambridge, UK) to determine whether human insulin could bind to freshly perfused adult *S. japonicum* worms washed 3 times with perfusion buffer. The HRP labelling was performed according to standard procedures as described above. The primary antibody solution was a 1:100 dilution of the mouse anti-human insulin monoclonal antibody and normal mouse serum (1:100) was used as a control. Non-specific antibody binding was inhibited by incubating the section in 10% (v/v) normal goat serum in PBS.

### Adenosine diphosphate (ADP) assays

To measure the level of hydrolysis of ADP by the schistosome ILP, ADP assay kits (Sigma-Aldrich) were used to measure ADP levels of recombinant schistosome ILPs after their interaction with different concentrations of ADP. ADP levels in various proteins (0.13 mg/ml) were determined following their incubation for 20 min with different concentrations of ADP (0, 5, 15, 30 μM). These proteins included: (i) r*Sj*ILP; (ii) r*Sm*ILP; (iii) *S. japonicum* tegumental protein as a positive control [31, 32], extracted from adult *S. japonicum* using the freeze/thaw/vortex method [33]; (iv) human insulin; and (v) *S. japonicum* Kunitz type protease inhibitor (r*Sj*KI-1) [34]. Human insulin and r*Sj*KI-1 were employed as negative controls, both of which were shown to have no ADP binding sites after analysis using the 3DLigandSite software.

### Binding between *Sj*ILP/insulin and *S. japonicum* insulin receptors

Binding assays were performed using the Octet Red system (FortéBio, Menlo Park, CA, USA) in 96-well microplates at 25 °C as described [18]. Briefly, assays were carried out by placing the Streptavidin Biosensors (FortéBio, Fremont, USA) in the microplate wells and measuring changes in layer thickness (in nanometers, nm) over time (in seconds). Protein/peptides were biotinylated using a NHS-PEO_4_-biotin kit (Thermo Scientific, Rockford, IL, USA) and then they were immobilised to the Streptavidin Biosensors. First, a duplicate set of sensors was rinsed in kinetic buffer (1 mM phosphate, 15 mM NaCl, 0.1 mg/ml BSA, 0.002% Tween-20) for 300 s which served as the baseline. Secondly, sensors were immobilised for 600 s with 200 μl culture medium containing biotinylated protein/peptide. Thirdly, sensors were washed in kinetic buffer for another 600 s. Finally, sensors were exposed to a series of diluted samples run in 200 μl volumes in the same assay. BSA was used as a negative control. The association of the two proteins was monitored for 1000 s followed by dissociation in the kinetic buffer for 1000 s. Data analysis from the FortéBio Octet RED instrument included a double reference subtraction. Sample subtraction was performed using the BSA as a reference control, and sensor subtraction was performed on all samples automatically using Octet User Software 7 [35].

Two binding assays were undertaken as follows:

(i) To test the binding ability between r*Sj*ILP and recombinant proteins coding the ligand domain of *Sj*IR1 (r*Sj*LD1)/ligand domain of *Sj*IR2 (r*Sj*LD2) [18]. Briefly, biotinylated r*Sj*ILP (150 ng/μl) was immobilised to the Streptavidin Biosensors as described above and then exposed to a series of dilutions of the r*Sj*LD1 or r*Sj*LD2 proteins (7, 14, 28, 56, 112 ng/μl), after washing in the kinetic buffer. The association of the two proteins was monitored followed by dissociation in the kinetic buffer as described above.
  Furthermore, to compare the binding affinity of *Sj*LDs to r*Sj*ILP/human insulin, biotinylated r*Sj*ILD1 (150 ng/μl)/*Sj*LD2 (100 ng/μl) was immobilized to the Streptavidin Biosensors, and then exposed to r*Sj*ILP and human insulin at 65 and 130 ng/μl, respectively, followed by dissociation by kinetic buffer. The association between the r*Sj*LDs and r*Sj*ILP was real-time monitored followed by dissociation in the kinetic buffer.
(ii) To compare the binding affinity of r*Sj*ILP to SjIR peptides (including analogues 1, 3, 13, 15, the first two derived from *Sj*LD1 and the latter two from *Sj*LD2) [36] which have been demonstrated as parasite-specific insulin binding epitopes with the strong binding ability to human insulin. The positions of the four analogues used in this study are provided in the Additional file 2: Figure S1. Peptides were synthesised and purified as previously described [36]. *Sj*ILP immobilized sensors were exposed to analogue 1 (1.85–11.7 μM), analogue 3 (0.5–1.85 μM), analogue 13 (4.1–15.3 μM) and analogue 15 (0.21–1.07 μM) followed by dissociation by kinetic buffer. The association between r*Sj*ILP and the different peptides was real-time monitored followed by dissociation in the kinetic buffer.

## Results

### Sequences of schistosome insulin-like peptides

Complete cDNA sequences obtained for schistosome ILPs comprised an open reading frame (ORF) of 390 bp encoding 130 amino acids in *S. japonicum* (*Sj*ILP), an ORF of 399 bp encoding 133 amino acids in *S. mansoni* (*Sm*ILP). The cDNA sequence for *Sh*ILP was predicted based on genomic data published recently for *S. haematobium* [9, 10]; it showed an ORF of 396 bp encoding 132 amino acids in *S. haematobium* (*Sh*ILP). *Sj*ILP shares 63% and 65% amino acid sequence identity with *Sm*ILP and *Sh*ILP, respectively. There is 79% amino acid sequence identity between *Sm*ILP and *Sh*ILP. *Sj*ILP shares only 18% amino acid identity with human insulin. Using the SignalP 4.1 server, we found *Sj*ILP contains a signal peptide (M1-E24), a B chain (I25-F75) and an A chain (F94-S129); *Sm*ILP has a B chain (M26-K78) and an A chain (F97-N133) but no predicted signal peptide; *Sh*ILP contains a signal peptide (M1-E24), a B chain (I25-L76) and an A chain (F116-N152).

Schistosome ILPs have a basic insulin structure (Additional file 3: Figure S2) containing an A peptide and B peptide linked by disulfide bridges. The structures show conserved cysteines in the A chain with the signature amino acid sequence CCCX(2)CX(8)C present.

*Sj*ILP, *Sm*ILP and *Sh*ILP have the same exon-intron arrangement whereby two exons are separated by a large intron of 8207 bp, 11,235 bp and 11,868 bp in *S. japonicum*, *S. mansoni* and *S. haematobium,* respectively.

The tertiary protein structures of *Sj*ILP, *Sm*ILP and *Sh*ILP were predicted using PHYRE2, and they share very similar structure as shown in Additional file 3: Figure S2, which is the predicted *Sj*ILP 3D structure. Schistosome ILPs contain 85–87% of Alpha helix and 12% transmembrane helix (Y15-T30 in *Sj*ILP, L15-D30 in *Sm*ILP, H15-T30 in *Sh*ILP). Of note, we found three predicted ADP binding sites located at HIS59, ARG62, and ARG120 residues in *Sj*ILP (Fig. 2b); *Sm*ILP has two ADP binding site located at ASN60 and ARG123 (Additional file 3: Figure S2), whereas no ADP binding sites were predicted in ShILP indicating *S. haematobium* may bind ADP in a different manner compared with the other two species.

### ADP assays

Adenosine diphosphate (ADP) is produced from adenosine triphosphate via the action of ATPases and plays a critical role in energy transfer reactions and is more stable than ATP.

**Fig. 1:**
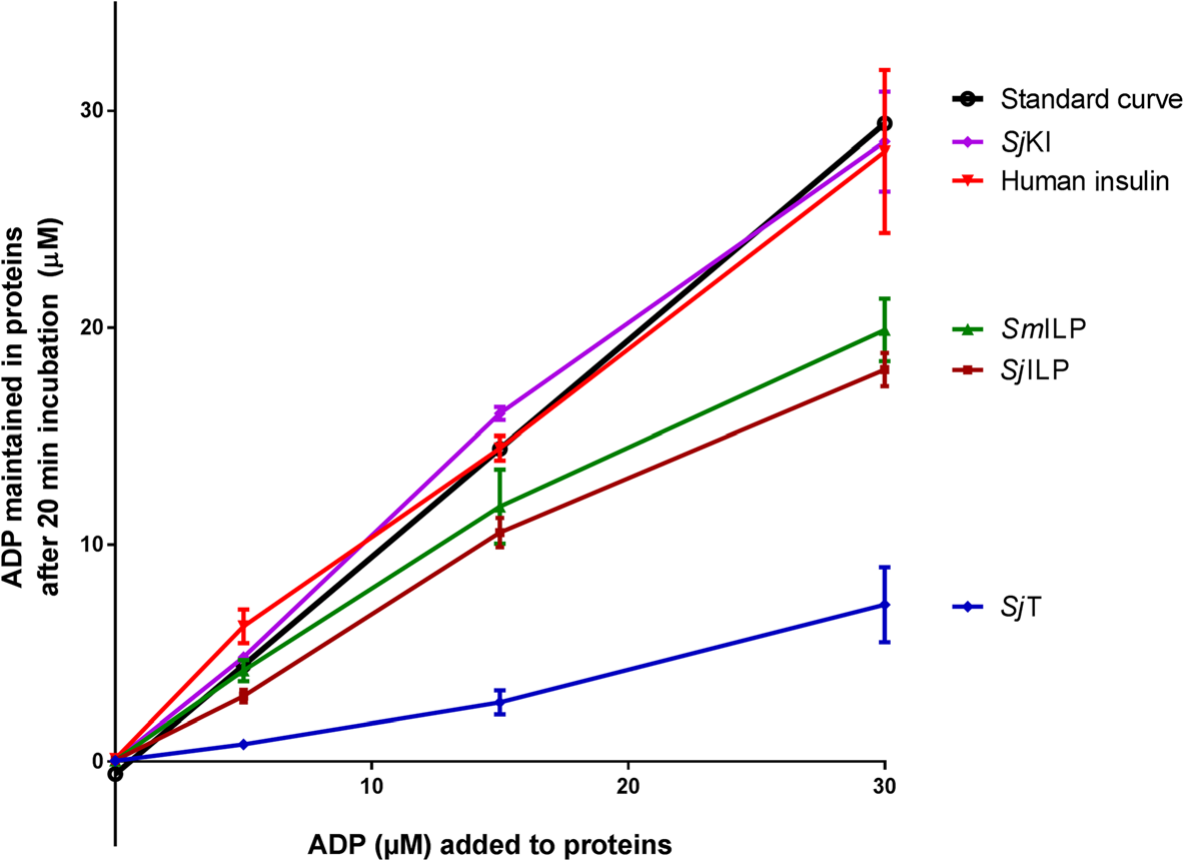
Adenosine diphosphate (ADP) assays with recombinant proteins (including *Sj*ILP and *Sm*ILP) and *S. japonicum* tegument protein. By using an ADP assay kit, ADP concentration (μM) was measured following incubation for 20 min with different proteins (0.13 mg/ml), including *Sj*ILP, *Sm*ILP, the positive control (adult *S. japonicum* worm tegument protein extract; *Sj*T) and negative controls (human insulin and r*Sj*KI-1). The assay kit ADP standard curve was obtained using different concentrations of ADP alone. Error bars represent the standard error of the mean (SEM). This experiment was performed three times

To determine the hydrolysis of ADP of schistosome ILPs, we used an ADP assay to measure the ADP levels maintained in proteins following their incubation for 20 min with different concentrations of ADP. Following incubation with ADP, the negative controls, human insulin and r*Sj*KI-1, produced, as expected, similar curves as the assay kit ADP standard curve (Fig. 1). The adult *S. japonicum* tegument protein extract hydrolysed 75–81% of ADP after incubation with ADP (5–30 μM), confirming the schistosome tegument possesses a protein/proteins which can hydrolyze ADP [32]. *Sj*ILP and *Sm*ILP consumed 29–39% and 21–34% of ADP, respectively. There was no significant difference in the levels of consumed ADP between *Sj*ILP and *Sm*ILP following their incubation with different concentrations of ADP, suggesting that both have similar ADP hydrolysis ability.

### Western blot analysis

**Fig. 2:**
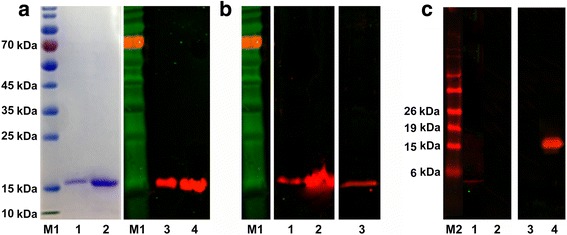
Western blot analysis using anti-*Sj*ILP serum to probe recombinant proteins (*Sj*ILP, *Sm*ILP and human insulin) and total extracts from adult schistosomes. **a** The SDS-PAGE gel of purified and refolded recombinant proteins *Sj*ILP (Lane 1) and *Sm*ILP (Lane 2). Mouse anti-*Sj*ILP antibody was used to probe r*Sj*ILP (Lane 3), r*Sm*ILP (Lane 4); **b** Mouse anti-*Sj*ILP antibody was used to probe carcass protein (Lane 1) and tegument protein (Lane 2) extracted from adult *S. japonicum,* and SWAP extracted from adult *S. mansoni* (Lane 3); **c** Mouse anti-human insulin monoclonal antibody was used to probe recombinant human insulin (Lane 1) and r*Sj*ILP (Lane 2). Mouse anti-*Sj*ILP antibody was used to probe recombinant human insulin (Lane 3) and r*Sj*ILP (Lane 4). Lanes M1 and M2, PageRuler pre-stained protein ladder in a different size. This experiment was performed three times

SDS-PAGE showed purified, and refolded r*Sj*ILP and r*Sm*ILP each migrated as a single band with the predicted sizes of approximately 15.6 kDa and 16 kDa, respectively (Fig. 2a). ELISA determinations indicated the titer of the mouse anti-*Sj*ILP serum used in the Western blot analysis was 1:25,600. Both r*Sj*ILP, r*Sm*ILP were recognised by the mouse anti-*Sj*ILP antibody (1:500) in a western-blot (Fig. 2a). Neither r*Sj*ILP nor r*Sm*ILP was recognised by naïve mouse sera (data not shown). Further, a band of approximately 16 kDa in extracts of both carcass protein (60 μg/well) and adult *S. japonicum* tegument protein (60 μg/well) was recognised by the anti-*Sj*ILP antibody (1:100) (Fig. 2b) with the signal being more pronounced in the latter. Cross immunological reactivity among the schistosome ILPs was observed in western blots using the anti-*Sj*ILP antibody (1:50), as it bound a band of approximately 16 kDa in SWAP from adult *S. mansoni* (45 μg/well) (Fig. 2b), thereby correlating well with the calculated molecular size of both ILPs; no bands were detectable when SWAP from either schistosome species was probed with naïve mouse serum (data not shown). Furthermore, no cross immunological reactivity was evident between *Sj*ILP, and recombinant human insulin was observed in western blots. Anti-*Sj*ILP antibody (1:500) only recognised r*Sj*ILP (20 μg/well), but did not bind to recombinant human insulin (20 μg/well), while anti-human insulin monoclonal antibody (1:50) only recognised human insulin (0.5 μg/well) at the expected molecular size of approximately 5.8 kDa, but did not react with r*Sj*ILP (0.5 μg/well).

To determine whether there was immunological cross-reactivity between r*Sj*ILP and human insulin, we used a mouse anti-human insulin monoclonal antibody and the mouse anti-*Sj*ILP antibody in Western blots to probe electrophoresed human insulin and r*Sj*ILP (Fig. 2). Whereas the anti-*Sj*ILP antibody bound r*Sj*ILP, it did not bind to human insulin, while the anti-human insulin antibody bound human insulin but not r*Sj*ILP.

**Fig. 3:**
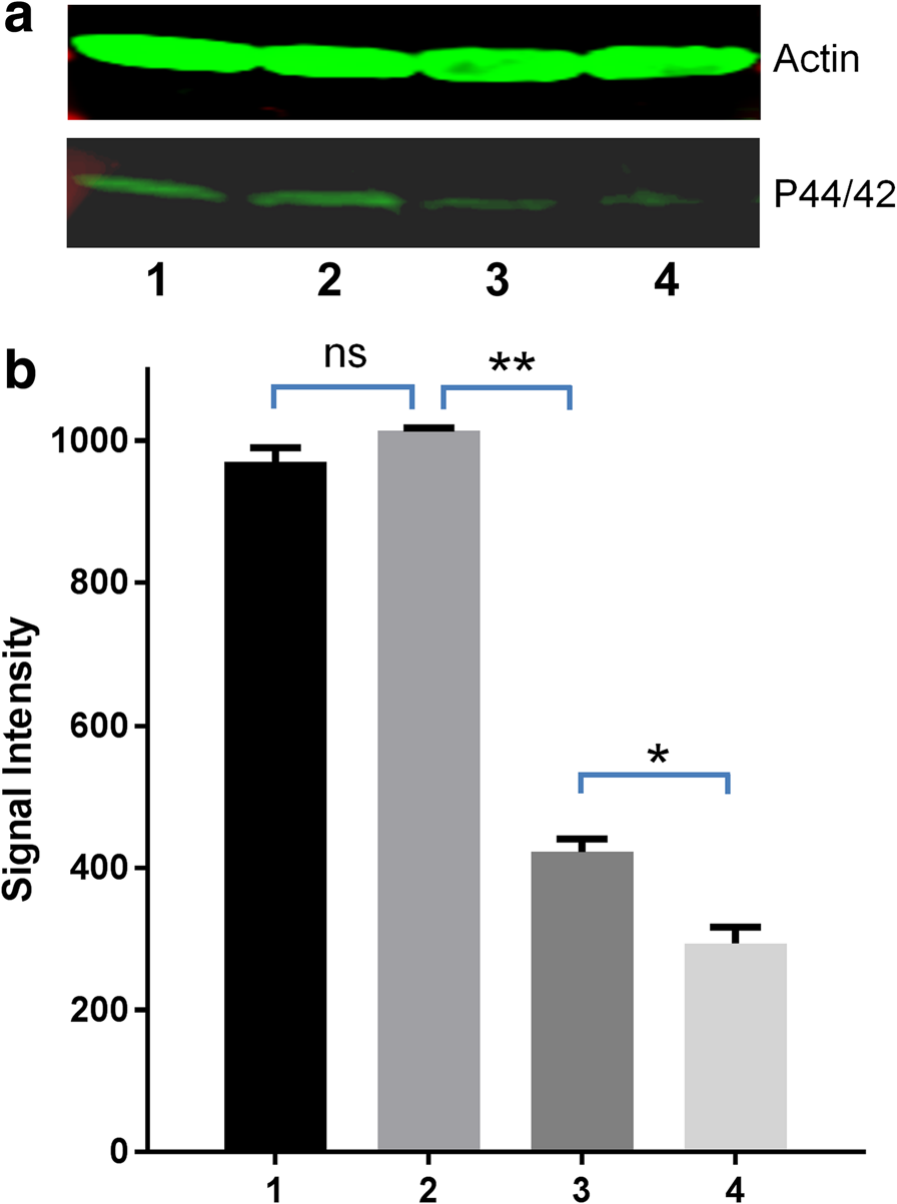
Effect of stimulation with r*SjI*LP or human insulin on extracellular signal-regulated kinase (Erk) in adult *S. japonicum* worms. **a** Anti-actin antibody (*upper*) and anti-phospho p44/42 MAPK (Erk) antibody (lower) were used to probe in Western blot analysis a protein extract of adult *S. japonicum* incubated for 30 min with: r*Sj*ILP (Lane 1); both r*Sj*ILP and human insulin (Lane 2); human insulin (Lane 3); neither r*Sj*ILP nor insulin (Lane 4). **b** Signal intensities of bands recognised by anti-phospho p44/42 MAPK (Erk) antibody in panel A (*lower*), were measured using the Odyssey Classic Infrared Imager with a scan intensity setting of 5 and sensitivity of 5. Error bars represent the standard error of the mean (SEM). This experiment was performed twice

An anti-phospho p44/42 MAPK antibody was used to detect the phosphorylation of Erk in adult *S. japonicum* stimulated by host insulin or r*Sj*ILP. An Erk band, approximately the same size (44 kDa) as observed previously in *S. mansoni* [37], was recognised by the anti-phospho antibody in protein extracts of adult *S. japonicum* worms incubated with or without r*Sj*ILP and insulin (Fig. 3). A 44 kDa Erk band was recognised in extracts prepared from worms stimulated with r*Sj*ILP and from worms co-stimulated with r*Sj*ILP and insulin, with no significant difference evident in band intensity between the two extracts (Fig. 3). The Erk band was also detectable in an extract from worms incubated with human insulin only but, compared with that of the worms co-stimulated with r*Sj*ILP and insulin, its intensity was reduced by 58% [*t*_(6)_ = 31.39, *P* = 0.001] (Fig. 3). The Erk band was detectable in an extract of worms incubated without human insulin and r*Sj*ILP, but its intensity was reduced by 30% [*t*_(6)_ = 4.353, *P* = 0.049] compared with that in the worm extract incubated with insulin only (Fig. 3) (*P ≤ 0.05, **P ≤ 0.001, ***P ≤ 0.0001).

### Distribution of *Sj*ILP and human insulin in adult *S. japonicum*

**Fig. 4:**
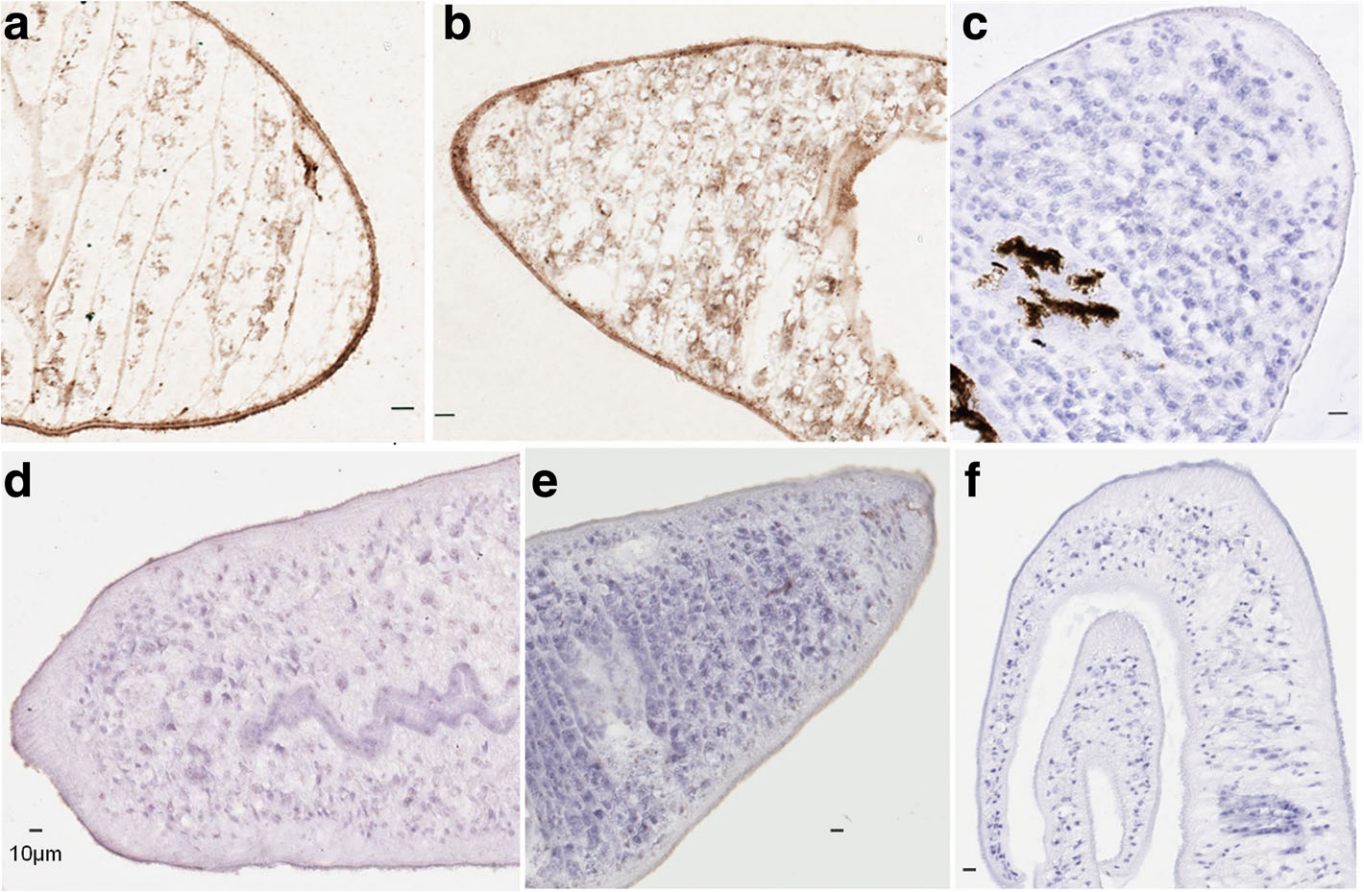
Immunolocalisation of *Sj*ILP and human insulin in adult *S. japonicum.* **a** Adult male and **b** female worm sections were labelled with mouse anti-*Sj*ILP antibody coupled with anti-mouse HRP and scanned using an Aperio scanner. **c** Negative control sections of the female worm were incubated with mouse pre-immune serum. **d** Adult male and **e** female worm sections were labelled with mouse anti-human insulin monoclonal antibody coupled with anti-mouse HRP and scanned using an Aperio scanner. **f** Negative control sections of the male worm were incubated with mouse pre-immune serum. *Scale-bars*: **a**-**f**, 10 μm

Indirect immunohistochemistry, incorporating HRP labelling (Fig. 4), indicated *Sj*ILP immunoreactivity occurred in the tegument, and the underlying musculature, similar to that observed previously for *Sj*IR1 [17] but was also present throughout the parenchyma of males and vitelline cells of females, the same locations as *Sj*IR2 [17]*.* No labelling was evident with control sera from pre-immunized mice.

To investigate further the interaction of human insulin with adult *S. japonicum*, we used HRP labelling to determine the distribution of human insulin in male and female worms (Fig. 4). Human insulin was detected only on the surface of adult male and female *S. japonicum,* co-located with *Sj*IR1 [17], probed using the anti-human insulin monoclonal antibody. No labelling was evident with control mouse serum.

### Binding assays

**Fig. 5:**
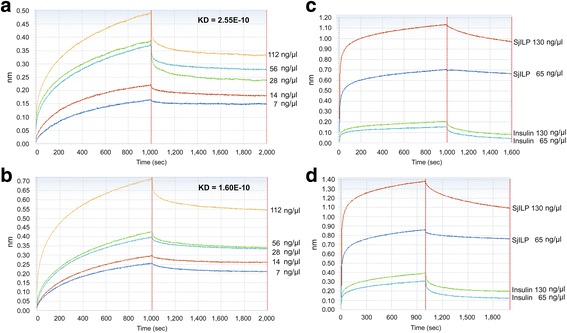
Binding affinity assays between *Sj*ILP/human insulin and *Sj*LD1/2 using the Octet RED system. The real-time binding response between r*Sj*ILP and (**a**) r*Sj*LD1, (**b**) r*Sj*LD2 at different protein concentrations (ng/μl) was measured in seconds (s). The parameters of the binding response (nm) and the KD value (M) of the binding between *Sj*ILP and *Sj*LD1/2 (7–112 ng/μl) are shown. **c** A comparison of the *Sj*LD1 binding ability to *Sj*ILP and human insulin at concentrations of 65 and 130 ng/μl. **d** A comparison of the *Sj*LD2 binding ability to *Sj*ILP and human insulin at concentrations 65 and 130 ng/μl. The coefficient of determination (*R* ^2^) for all the interactions was close to 1.0, indicating a good curve fit

**Fig. 6:**
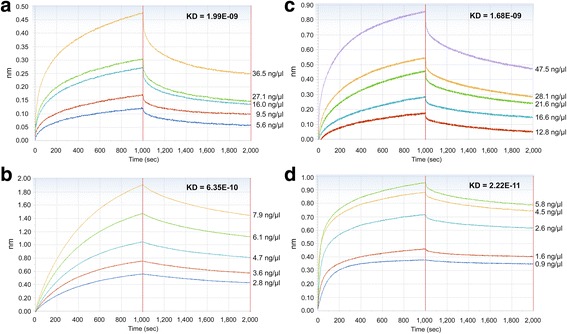
Binding affinity assays between *Sj*ILP and analogues 1, 3, 13, 15 derived from *Sj*LDs, respectively, using the Octet RED system. The binding response between r*Sj*ILP and analogues (**a**) 1, (**b**) 3, (**c**) 13, (**d**)15 at different protein concentrations (μg/ml) was measured in seconds (sec). The parameters of the binding response (nm) and the KD value (M) of the binding affinity between *Sj*ILP and peptides at different concentrations are shown. The coefficient of determination (*R* ^2^) for all the interactions was close to 1.0, indicating a good curve fit

Octet RED technology was used to measure the binding affinity between *Sj*ILP and *Sj*LDs and between *Sj*ILP and peptides, including analogues 1, 3 derived from *Sj*LD1, and analogues 13, 15 derived from *Sj*LD2 [36]. Our previous study showed that analogues 1, 3, 13 and 15 are mainly responsible for host insulin binding affinity in *S. japonicum* [36]*.* Interactions between r*Sj*ILP and *Sj*LDs are presented in Fig. 5; there was strong interaction in vitro between r*Sj*ILP and r*Sj*LD1 [KD = 2.255e^-10^, coefficient of determination (*r* ^2^) = 0.99] and r*Sj*ILP and r*Sj*LD2 (KD = 1.6e^-10^, *r* ^2^ = 0.96) detected in the protein concentration range 7–112 μg/ml. Increasing the concentration of *Sj*LD1/2 led to an increased binding response with the dissociation phase slowly decreasing. To compare the binding affinity of the *Sj*LDs to *Sj*ILP and human insulin, *Sj*LD1/2 immobilised sensors were exposed to *Sj*ILP or insulin at 65 and 130 ng/μl, respectively. At the same protein concentration, *Sj*ILP exhibited 4.6–5.5 times stronger binding to *Sj*LD1 (Fig. 5c) and 2.8–3.5 times stronger binding to *Sj*LD2 (Fig. 5d) than human insulin.

r*Sj*ILP strongly bound analogues 1 and 3, exhibiting KD values of 1.99e^-09^ (*r* ^2^ = 0.92, in the peptide concentration range 5.6–36.5 ng/μl) and 6.35e^-10^ (*r* ^2^ = 0.99, in the peptide concentration range 2.8–7.9 ng/μl), respectively (Fig. 6a, b). *Sj*ILP was also able to bind analogues 13 and 15 with KD values of 1.68e^-09^ (*r* ^2^ = 0.93, in the peptide concentration range 12.8–57.5 ng/μl) (Fig. 6c) and 2.22e^-11^ (*r* ^2^ = 0.98, in the peptide concentration range 0.9–5.8 ng/μl) (Fig. 6d), respectively.

## Discussion

It has been demonstrated that two types of insulin receptors (IR-1 and IR-2) from *S. japonicum* [17], *S. mansoni* [16], and *E. multilocularis* [38] can bind to human insulin, thereby activating the insulin signalling pathway which plays an important role in the regulation of worm glucose uptake, adult worm fecundity and the differentiation of germline stem cell populations [16, 17, 38].

Genome-wide searches have recently identified insulin-like peptides (ILPs) in parasitic flatworms [20], including schistosomes, but no further studies on blood fluke ILPs have been carried out. The ILPs, which initiate insulin signalling by binding to their insulin receptors, are a large multigene family that have been characterised extensively from invertebrates [39, 40]. To better understand the characterisation of ILPs in schistosomes, we isolated the ILP genes from *S. japonicum* (*Sj*ILP) and *S. mansoni* (*Sm*ILP) and showed they exhibit 63% protein sequence similarity and a high level of cross immunological reactivity observed in western blots using an anti-*Sj*ILP antibody. Schistosome ILPs may possess conserved features shared by the members of the insulin/insulin-like family, including tertiary structures comprising 85–87% alpha and 12% transmembrane helices; a gene structure featuring two small exons separated by a much larger sized intron; conserved splicing sites, and six strictly conserved cysteines forming three disulfide bonds. The disulfide bonds are predicted to form two inter chain bridges across the two chains and one intra-chain bond on the A chain, which is deemed to be pivotal for the acquisition of the functional structure of the mature dimeric peptide [41].

We were able to confirm that both *Sj*ILP and *Sm*ILP can bind ADP, as predicted using PHYRE2, whereas, no ADP binding ability was observed with human insulin, suggesting a specific role for ILPs in schistosomes. It has been demonstrated previously that schistosome tegumental extracts possess ATP and ADP hydrolysing activity [31, 32] and ATP hydrolysis commonly leads to the generation of ADP, which is a major agonist of the recruitment and aggregation of platelets [42]. Further studies discovered that the binding or hydrolysis of exogenous ADP led to the inhibition of platelet aggregation and thrombus formation around the worms [43]. It is known that the peptide growth factor insulin-like growth factor (IGF)-1, which is structurally related to insulin, can affect ADP-ribosylation processes and interactions with glucocorticoids which are important in the regulation of glucose metabolism during the maturation and differentiation of astroglial cells [44, 45]. Based on our ADP assays showing *Sj*ILP hydrolysed 29–39% of ADP compared with human insulin, we hypothesis that *Sj*ILP, with common conserved signatures shared with members of the insulin/insulin-like family, may also function in a similar role as IGF-1 after binding to exogenous ADP, but this requires further in vivo experimental investigation.

By sharing the same binding epitopes (analogues 1, 3 and 13, 15) [18], *Sj*ILP has a stronger bind affinity with the *Sj*IRs than human insulin. Our previous research and that of others have shown that human insulin can stimulate the schistosome insulin pathway by binding parasite IRs and activating the downstream Erk/MAPK and PI3K/AKt sub-pathways [15], thereby playing an essential role in worm growth and development [46]. To further determine if the binding between r*Sj*ILP/human insulin and *Sj*IRs could activate downstream Erk/MAPK signal transduction, we used an anti-phospho antibody against Erk to detect the phosphorylation of Erk in adult *S. japonicum* following incubation with host insulin/r*Sj*ILP. We found that following stimulation with either r*Sj*ILP or human insulin, adult *S. japonicum* was able to induce the phosphorylation of Erk. However, a weak phosphor-p44 band was also observed in the SWAP of *S. japonicum* cultured in DMEM without insulin/r*Sj*ILP, indicating ILP expressed by the worm itself can activate Erk. In addition, more phosphorylated Erk was observed in worms stimulated with human insulin compared with worms cultured without insulin, further supporting our previous report that by binding to *Sj*IR, host insulin can activate the Erk/MAPK sub-pathways in schistosomes. [15]. We found Erk was strongly phosphorylated in worms stimulated with exogenous r*Sj*ILP or co-stimulated with *Sj*ILP and insulin, compared with those incubated with human insulin alone. There was no significant difference between worms treated with r*Sj*ILP and co-treated with r*Sj*ILP and human insulin, suggesting that *Sj*ILP binds *Sj*IRs and activates downstream signalling more strongly than human insulin; this may due to the stronger binding of *Sj*LD*-Sj*ILP compared to that of *Sj*LD-human insulin (Fig. 5).

The immunolocalisation analysis showing that *Sj*ILP is distributed on the worm tegument and in the parenchyma/vitelline tissue of adult *S. japonicum* matches the western blot results indicating that native *Sj*ILP can be recognised by the anti-*Sj*ILP antibody both in the *S. japonicum* tegument and in carcass protein (Fig. 2b). Furthermore, the co-distribution of *Sj*ILP and *Sj*IR1 on the worm tegument and same location of *Sj*ILP and *Sj*IR2 in the parenchyma/vitelline tissue in adult parasite provides are suggestive of an interaction between the schistosome ILP and *Sj*IR1 and 2, respectively, in different locations in *S. japonicum*. The immunolocalization study showing human insulin was detected on the tegument of adult worms with less HRP labelling compared to that observed in *Sj*ILP, supports the results of our binding assays showing the *Sj*ILP has a stronger bind affinity with *Sj*LD than with human insulin. In addition, the distribution of human insulin only on the surface of worms, indicates that it may be able to bind *Sj*IR1 on the surface with no opportunity to cross though the tegument and bind to *Sj*IR2 inside the parasite, although human insulin has been shown to bind strongly with both *Sj*IR1 and *Sj*IR2 which share similar binding epitopes [18]. Compared with *Sj*IR2, *Sj*IR1 may be more involved in utilising host insulin, having a specialised function in the parasite to exploit the mammalian host hormone, with the IR-1 homologue having been lost by other taxa during evolution [17]. Given the important role of insulin in glucose uptake in *S. japonicum* and the same location of *Sj*ILP/human insulin and SjIR1 on the surface of adult worms leads to a logical hypothesis that the transport of glucose from host blood into schistosomes may be regulated by the binding between *Sj*ILP/human insulin and *Sj*IR1 occurring at the parasite surface although this requires further verification. Once glucose is taken up into the schistosome worm, it would be transferred into different tissues and cells with this process being regulated by the binding between *Sj*ILP and *Sj*IR2. Both molecules are co-located in the parenchyma of males and the vitelline cells of females and play important roles in the regulation of growth, adult fertility and the differentiation of germline stem cell populations [15, 18, 20].

## Conclusions

The findings presented here shed new light on the function and utilisation of ILPs in schistosomes and provide further insight on the distinct and specialised functions of *Sj*IR1 and 2 in *S. japonicum* and their interaction with host insulin.

## Additional files

Additional file 1: Table S1. Primers used in PCR to obtain full-length cDNA sequences encoding schistosome insulin-like peptides in *S. japonicum (Sj*ILP) *S. mansoni* (*Sm*ILP). (DOC 28 kb)

Additional file 2: Figure S1. Alignment of amino acid sequences of extracellular regions of different insulin receptors using CLUSTAL W. The extracellular regions of *Sj*IR1 and 2 were aligned with those from insulin receptors in *Homo sapiens* (HIR) and *Drosophila melanogaster* (DmIR). Black boxes indicate identical amino acids and grey boxes denote sequence similarity. Peptides P13, P1, are boxed in red and P15, P3 are shown in green. These peptides all bound *Sj*ILP or human insulin using the Octet RED system. *Sj*LD1 contains amino acid sequence from D59-E411 in *Sj*IR1 and *Sj*LD2 contains sequence from R37-C525 in *Sj*IR2. (DOC 56 kb)

Additional file 3: Figure S2. (**A**) Schematic representation of the predicted structures of insulin-like peptides in *Schistosoma japonicum* (*Sj*ILP), *S. mansoni* (*Sm*ILP) and *S. haematobium* (*Sh*ILP). Schistosome ILPs contain an A peptide (thick underline) and a B peptide (double underline) linked by disulfide bridges; a signal peptide was predicted only in *Sj*ILP and *Sh*ILP (boxed in green). The conserved cysteines in the A chain with the amino acid sequence CCCX(2)CX(8)C are indicated in dark red. The putative disulfide bonds between conserved cysteines are indicated by the S-S bridge in red. (**B**) Predicted tertiary protein structures of schistosome ILPs and the location of adenosine diphosphate (ADP) binding sites. (**a**) Three-dimensional model of *Sj*ILP determined using Phyre2. Image coloured by rainbow from N to C terminus, Model dimensions (Å): X:28.233, Y:29.795, Z:26.620) are the same as those of *Sm*ILP and *Sh*ILP. (**b**) Three predicted ADP binding sites of *Sj*ILP located at HIS59, ARG62, and ARG120 residues. (**c**) Two ADP binding sites of *Sm*ILP presented at ASN60 and ARG123. No ADP binding sites were predicted in *Sh*ILP. (TIF 1820 kb)

## Abbreviations

ADP: Adenosine diphosphate
ELISA: Enzyme-linked immunosorbent assay
Erk: Extracellular signal–regulated kinase
GTP4: Glucose transport protein 4
GYS: Glycogen synthase
HRP: Horseradish peroxidise
ILP: Insulin-like peptide
IR: Insulin receptor
LD: Ligand domain of insulin receptor
MAPK: Mitogen-activated protein kinase
OCT: Optimal cutting temperature
PI3K: Phosphatidylinositide 3-kinase
RTKs: Receptor tyrosine kinases
SHC: Src homology-containing
SWAP: Soluble adult worm antigen preparation
