# Supplementary material for: Identification and functional characterisation of a Schistosoma japonicum insulin-like peptide
Source: Parasit Vectors. 2017 Apr 14;10:181. doi: 10.1186/s13071-017-2095-7 (PMC5391603; doi:10.1186/s13071-017-2095-7)
Supplement: Supplementary file 1 — Primers used in PCR to obtain full-length cDNA sequences encoding schistosome insulin-like peptides in S. japonicum (SjILP) S. mansoni (SmILP). (DOC 28 kb) [file 13071_2017_2095_MOESM1_ESM.doc]

**Additional file 1: Table S1.** Primers used in PCR to obtain full-length cDNA sequences encoding schistosome insulin-like peptides in *S. japonicum (Sj*ILP) and *S. mansoni* (*Sm*ILP)

| ID of primers | Primer pair sequence (5ˈ-3') | | |
| --- | --- | --- | --- |
|  | Forward | Reverse | Size (bp) |
| *Sj*ILP | ATGAGAATATTATTTTGGTTAATGTTAACCATAC | TAGCTAGGATTGCAAAATTGTTCT | 390 |
| *Sm*ILP | ATGAAAAAATTTTTATTTTCCTTCATAGTAACAATTTTCTGTT | ATTTGGATTACAAAATTGTTCTAGATAGTTACGATTACAA | 399 |
