# Supplementary material for: Identification and functional characterisation of a Schistosoma japonicum insulin-like peptide
Source: Parasit Vectors. 2017 Apr 14;10:181. doi: 10.1186/s13071-017-2095-7 (PMC5391603; doi:10.1186/s13071-017-2095-7)
Supplement: Supplementary file 2 — Alignment of amino acid sequences of extracellular regions of different insulin receptors using CLUSTAL W. The extracellular regions of SjIR1 and 2 were aligned with those from insulin receptors in Homo sapiens (HIR) and Drosophila melanogaster (DmIR). Black boxes indicate identical amino acids and grey boxes denote sequence similarity. Peptides P13, P1, are boxed in red and P15, P3 are shown in green. These peptides all bound SjILP or human insulin using the Octet RED system. SjLD1 contains amino acid sequence from D59-E411 in SjIR1 and SjLD2 contains sequence from R37-C525 in SjIR2. (DOC 56 kb) [file 13071_2017_2095_MOESM2_ESM.doc]

SjIR_2 -------------------------------MLNILAQHDSTDLG--------------------------------SFQEVACTR--ADVRHSSS-LTKLSRCTVIEGDLFIVFTR--- 51 **P13**

SjIR_1 MQTTHPQTIRTADLVVCTYSEINASSMVPRFALVICTPHVIVSLHPNLRN------------------------VFPNHHCKDCSGRLLNLRESNN-LSSLTNCSTIHGTLVIRNLDDSC 95 **P1**

HIR -------------MGTGGRRGAAAAPLLVAVAALLLGAAGHLYPG------------------------------------EVCPG--MDIRNNLTRLHELENCSVIEGHLQILLMFKTR 69

DmIR(259) RHKCFHYKHNYSYSPGISLLLFILLANTLAIQAVVLPAHQQHLLHNDIADGLDKTALSVSGTQTRWPRSESNPTMRLSQNVKPCKS--MDIRNMVSHFNQLENCTVIEGFLLIDLIN--- 374

**L1 subdomain**

SjIR_2.1 --IPRDASLPFLKEVTGSLLVYDTEGPEDLSILLPNLTLVR-----------GQTLVFGYSVVIKSTS-FKSIGLPSLRVIQQGGVRIDSNPQLCYVETIDWKVILQNQS-GDVSSIKIV 156 **P15**

SjIR_1.1 -CLSINCSLPNVVEITGSLIIENGNCSGDLSTLLPNLTVIRNQVIPSTEHNYGNSQISDYSLIIRHTK-LKGIGLWKLKTLNSYPIALIDNPLMCFVDTVNWNALISTPTTGSIMSYRSV 213 **P3**

HIR PEDFRDLSFPKLIMITDYLLLFRVYGLESLKDLFPNLTVIR-----------GSRLFFNYALVIFEMVHLKELGLYNLMNITRGSVRIEKNNELCYLATIDWSRILDSVEDNYIVLNKDD 178

DmIR DASPLNRSFPKLTEVTDYIIIYRVTGLHSLSKIFPNLSVIR-----------GNKLFDGYALVVYSNFDLMDLGLHKLRSITRGGVRIEKNHKLCYDRTIDWLEILAENETQLVVLTENG 483

**CR**

SjIR_2.1 KNGLICSNICSSSCSVNSPNNTQHSSVELWRSPFSDILPTDGHCWSMNECQSICPVYCTLQNLTCTMKRPYKCCHPECLAGCYGDG--PSECVACKNVMHDNQCISKCPSGTFKYLNRRC 274

SjIR_1.1 REQ-LVRFNLGNFCPD-------HRSANCFS-YPDDSSRSS--CWSANSCQAKCSSVCTVNGLPCYLNDPEKCCDSECSGGCSGPS--PSDCLSCKHVNLNGTCLSHCPSSYYLLNNLYC 320

HIR NEE------CGDICPG-----TAKGKTNCPATVINGQFVER--CWTHSHCQKVCPTICKSHG--CTAEG--LCCHSECLGNCSQPDD-PTKCVACRNFYLDGRCVETCPPPYYHFQDWRC 280

DmIR KEKECRLSKCPGEIRIEEG--HDNTAIEGELNASCQLHNNRRLCWNSKLCQTKCPEKCRNN---CIDEH--TCCSQDCLGGCVIDKNGNESCISCRNVSFNNICMDSCPKGYYQFDS-RC 595

SjIR_2.1 VTEYQCLVELPNMMYSDSPYISEYYAN----NSFP---YNDMTRMHSNFTIFNGSCLLKCPVGYKKSLKTGHCYACGD---QCELKHCREFLIYNLKALDTLTGCYSAKA---IYLSIQD 381

SjIR_1.1 IARDECINRQEIIEN----VHGNYLAN---------------------YSIFNFTCILKCPLHYVR-SVSGECQFRPDS--KFLKRACGDINMYKVSDLKQVRGCVTAQS---ILISIRD 409

HIR VNFSFCQDLHHKCKN------SRRQGC-----------H--------QYVIHNNKCIPECPSGYTMNSSNLLCTPCLGPCPKVCHLLEGEKTIDSVTSAQELRGCTVING---SLIINIR 372

DmIR VTANECITLTKFETN------SVYSG-----------------------IPYNGQCITHCPTGYQKSENKRMCEPCPG---GKCDKECSSGLIDSLERAREFHGCTIITGTEPLTISIKR 683

**L2 subdomain**

SjIR_2.1 GKPETVANLLDRAFKNLKVIHHSLRIVRSSVLENLNFLQHVHSIGQIG--ENVSSHP--IVFELFGNDNLRNLWPIAKDANNIEDDPVNSLQILSNGLIRITQNRQLCPEKIFQLFESNI 497

SjIR_1.1 CEDADTSEFMD-AFSDLEEIYTSLHVISSDSLQSLAFLRSLRVIHGLNRDGTVPSGSRKTVLEIAWNSQLKSLWLPVSSTLIIQH-----GRVMFT------LNRNLCPNDVKKFINFN- 516

HIR GGNNLAAELEA-NLGLIEEISGYLKIRRSYALVSLSFFRKLRLIRGET----LEIGN--YSFYALDNQNLRQLWDWSKHNLTITQ-----GKLFFH------YNPKLCLSEIHKMEEVS- 473

DmIR ESGAHVMDELKYGLAAVHKIQSSLMVHLTYGLKSLKFFQSLTEISGDP-----PMDADKYALYVLDNRDLDELWGPNQTVFIRKG------GVFFH------FNPKLCVSTINQLLPML- 785

**FnIII-1**

SjIR_2.1 IKSSGNNNITLTQAEREMITITNGDLAYCNWREFNVTVSNRSSHSVRVTWPHPSTIHFTNKSVNTNFSDGSNSSHVDEVVLIYLFYQSAPNDLPDIIKGRRVYDKNSWRMLTTSCDLNPG 617

SjIR_1.1 VNLSRNL----SNLEFDLIEKSNGAIGLCRTHRLNLSMN----------YVYQRSISFT--ITNTPAWNDLRQILPTTVYYRYIGIDESESIIETICDKSWHIHEPKCHKVIEPITNMDF 620

HIR --GTKGR-----QERNDIALKTNGDQASCENELLKFSYIR---------------TSFD---KILLRWEPYWPPDFRDLLGFMLFYKEAPYQNVTEFDGQDACGSNSWTVVDIDPPLRSN 568

DmIR --ASKPK----FFEKSDVGADSNGNRGSCGTAVLNVTLQS---------------VGAN--SAMLNVTTKVEIGEPQKPSNATIVFKDPRAFIGFVFYHMIDPYGNSTKSSDDPCDDRWK 882

**FnIII-1 FnIII-2**

SjIR-2 DSISSFSYLELDCGLTLSELEAATRYAIYVEMKFPLKQT----GAVSNLVYFTTLSVNPTPPQYPWLEPLNQNSLRLTWMPPVKPSGIIDAYLIWIRILEDNPSEYLTQDFCTHRPNWIQ 733

SjIR-1 FHGTQSSGLKLHCEVNY--LNPAQRYQAYVEIRTMFNSE----GALSQVFTFQTKQDKPSSPTDFRAYPLNSNKIQLYWNSPDNPNGQIVEYHVWYRRLSLNVSSFGDGAFCTDGRVSSR 734

HIR D--P---KSQNHPGWLMRGLKPWTQYAIFVKTLVTFSDERRTYGAKSDIIYVQTDATNPSVPLDPISVSNSSSQIILKWKPPSDPNGNITHYLVFWERQAED-SELFELDYCLKGLKLPS 682

DmIR VSSP-----EKSGVMVLSNLIPYTNYSYYVRTMAISSELT---NAESDVKNFRTNPGRPSKVTEVVATAISDSKINVTWSYLDKPYGVLTRYFIKAKLINRP-TRNNNRDYCTEPLVKAM 993

Insert **FnIII-2**

SjIR-2 SGWDKNSLFKHSRQINLH------------NDYCDSCLPCPNLLEIEQLSNTQAIPSLDK----NYWPPSVSRWMTDNFGNILPV-IVDEV-VSLKSDTVGLIVQKNASTFQHISHFQMD 835

SjIR-1 LMSLPTMSNGNTVNNLPQHSTSYVNLSKTIKRICSCTSCTAFCIKPSVQLEKTNNGGRNTLLRLNTIQGSIFNGRPSNDNHYGSDYEKLTRFECLDMIRFEDSLQNLLLFSRIKSPLKIR 854

HIR RTWSPPFESEDSQKHNQS------------EYEDSAGECCSCPKTDSQILK----------------ELEESSFRKTFEDYLHNVVFVPRKTSSGTGAEDPRPSRK----RRSLGDVGNV 770

DmIR ENDLPATTPTKKISDPLAGD---------CKCVEGSKKTSSQEYDDRKVQAGMEFENALQNFIFV-PNIRKSKNGSSDKSDGAEGAALDSNAIPNGGATNPSRRRRDVALEPELDDVEGS 1103

**FnIII-2** **FnII-3**

SjIR-2 YEKK-CIHSVNENVKYLTGFPPFTRVLVEIQACLSYDSISYVES-ETKC------HRPPPWSNLKSGTTIYLHQLHHQWSVCEYE------------------------LCSPRSTVINR 923

SjIR-1 RKRNIRSNFLDFLNSKEIEQHHHHYFSREWDGHLRISANPNIIGNHELINN----TVSGVLIDGLHHYSEYLFVISACHSPHDINGEPLITSNTTFFNDNDDNLVSDMPWCSSRNVVWQR 970

HIR TVAVPTVAAFPNTSSTSVPTSPEEHRPFEKVVNKESLVISG-----------------------LRHFTGYRIELQACNQDTPEE------------------------RCSVAAYVSAR 843

DmIR VLLRHVRSITDDTDAFFEKDDENTYKDEEDLSSNKQFYEVFAKELPP--------NQTHFVFEKLRHFTRYAIFVVACREEIPSEKLRD--------------TSFKKSLCSDYDTVFQT 1201

**FnIII-3**

SjIR-2 VNPHVESDKVPVASIHAT-----------------------------------------------------LNGPGSIRITWSNPIKPNGLILHYLLRYR-----------------PRN 973

SjIR-1 TEASIGVDDVDSDSIQLIDENIDTNICLSSS-ETSLSGLMKQEANLCNKMFNFSSIDFSESNHTRIGK---SSKTTAKRLQWSPPLQPNGLTLYYWIRYRRLD---------------VE 1071

HIR TMPEAKADDIVGPVTHEI-----------------------------------------------------FEN-NVVHLMWQEPKEPNGLIVLYEVSYR-------------------- 889

DmIR TKRKKFADIVMDLKVDLEHA---------------------------------------------------NNTESPVRVRWTPPVDPNGEIVTYEVAYK-------------------- 1250

**FnIII-3**

SjIR-2 HDQSYTDS--NHSSSDVSLPWLTKCISMSHWSADHSEH-A----------LTSSSYITINQKEVSRSKRGY--------------NANSSTTDG---GISIKDLSPGSYEFQILAVSLAG 1063

SjIR-1 LKSKKLSSTVS---THEQ-AWSVICLNAINLSNSINEN----------------------------------------------------TSANRFVSVQLYDLRSGVYEFQIMSVSLSG 1135

HIR ---------------RYGDEELHLCVSRKHFALER--------------------------------------------------------------GCRLRGLSPGNYSVRIRATSLAG 932

DmIR -------------LQKPDQVEEKKCIPAADFNQTAG---------------------------------------------------------------YLIKLNEGLYSFRVRANSIAG 1294

SjIR-2 NGEWSPTVIFNIPFYTDHNGTINRQK--- 1089

SjIR-1 NGSWTSLKQFKVFDTSVHTTWGFLEEHYY 1181

HIR NGSWTEPTYFYVTDYLDVPSN-------- 953

DmIR YGDFTEVEHIKVEPPPSY----------- 1312
