# Supplementary figures and images for: Identification and functional characterisation of a Schistosoma japonicum insulin-like peptide
Source: Parasit Vectors. 2017 Apr 14;10:181. doi: 10.1186/s13071-017-2095-7 (PMC5391603; doi:10.1186/s13071-017-2095-7)

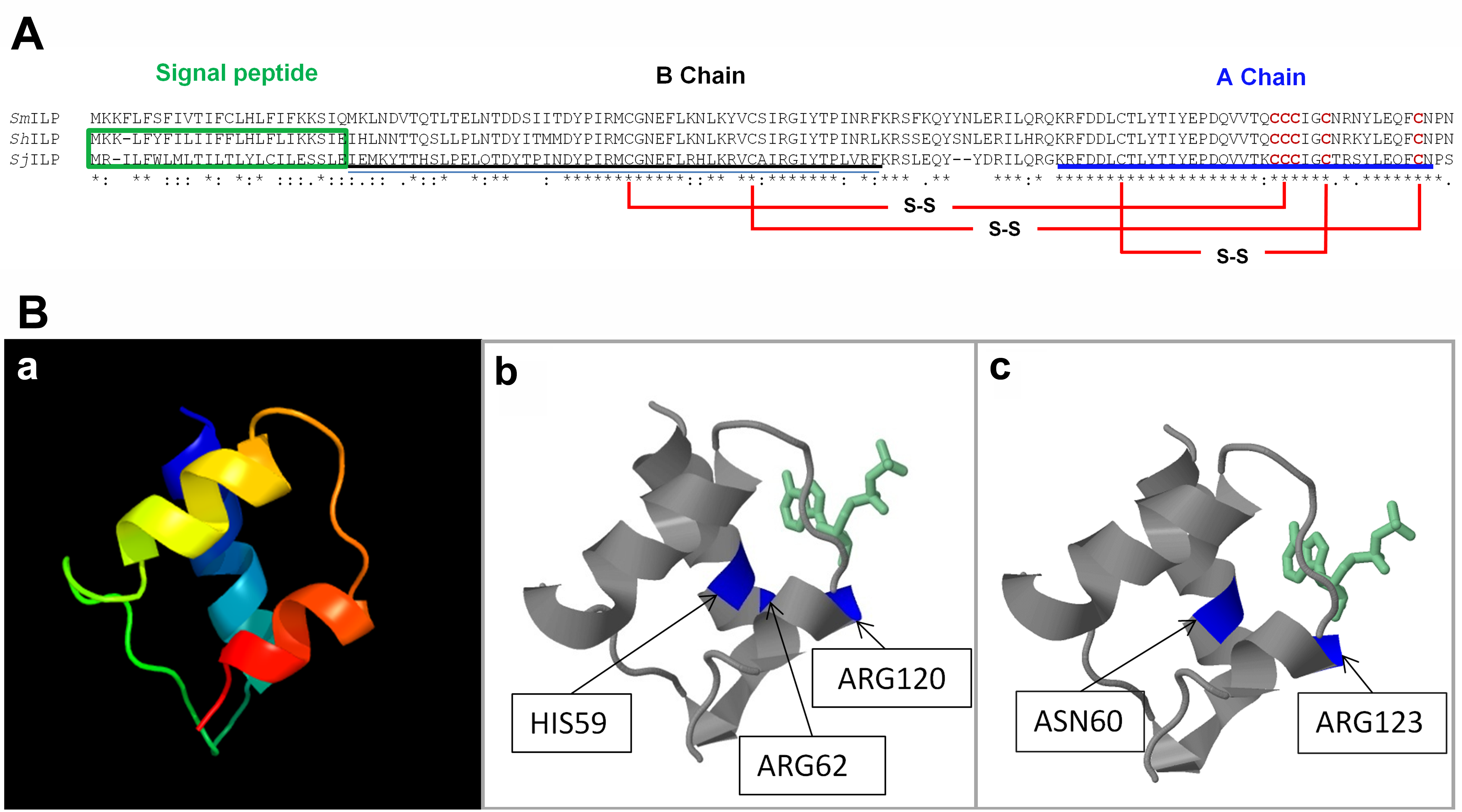

Supplement: Supplementary file 3 — (A) Schematic representation of the predicted structures of insulin-like peptides in Schistosoma japonicum (SjILP), S. mansoni (SmILP) and S. haematobium (ShILP). Schistosome ILPs contain an A peptide (thick underline) and a B peptide (double underline) linked by disulfide bridges; a signal peptide was predicted only in SjILP and ShILP (boxed in green). The conserved cysteines in the A chain with the amino acid sequence CCCX(2)CX(8)C are indicated in dark red. The putative disulfide bonds between conserved cysteines are indicated by the S-S bridge in red. (B) Predicted tertiary protein structures of schistosome ILPs and the location of adenosine diphosphate (ADP) binding sites. (a) Three-dimensional model of SjILP determined using Phyre2. Image coloured by rainbow from N to C terminus, Model dimensions (Å): X:28.233, Y:29.795, Z:26.620) are the same as those of SmILP and ShILP. (b) Three predicted ADP binding sites of SjILP located at HIS59, ARG62, and ARG120 residues. (c) Two ADP binding sites of SmILP presented at ASN60 and ARG123. No ADP binding sites were predicted in ShILP. (TIF 1820 kb) [file 13071_2017_2095_MOESM3_ESM.tif]
